# Analysis of the Response of Prostate Cancer to Ultra-Hypofractionated High-Dose-Rate Brachytherapy: The Role of Hypoxia and Reoxygenation

**DOI:** 10.3390/cancers18122007

**Published:** 2026-06-21

**Authors:** Eva G. Kölmel, Pedro Otero-Casal, Juan Pardo-Montero

**Affiliations:** 1 Group of Medical Physics and Biomathematics, Instituto de Investigación Sanitaria de Santiago (IDIS), 15706 Santiago de Compostela, Spain; 2 Department of Medical Physics, Complexo Hospitalario Universitario de Santiago de Compostela, 15706 Santiago de Compostela, Spain; 3 Department of Particle Physics, Universidade de Santiago de Compostela, 15782 Santiago de Compostela, Spain

**Keywords:** prostate cancer, HDR-BT, radiobiological modeling, LQL model, hypoxia, reoxygenation

## Abstract

**Background/Objectives:** Clinical studies of prostate cancer treated with radically hypofractionated high-dose-rate brachytherapy (HDR-BT) have reported a significant loss of tumor control that contradicts the standard linear-quadratic (LQ) and low-α/β-ratio paradigm for prostate cancer. In a previous study by our group, we showed that the linear–quadratic–linear (LQL) model could describe this response, but the underlying biological drivers remained unclear. In this follow-up study, we further investigate whether the interplay between hypoxia and reoxygenation kinetics can explain the poor response to extreme hypofractionation. **Methods:** We analyzed a large dataset of 3239 patients (44 schedules) using a three-compartment reoxygenation model (the MSK model) that simulates the dynamics of oxic, intermediate, and hypoxic cell populations. **Results:** The results show that the MSK model achieves an excellent fit to the clinical data (p>0.99) while maintaining a biologically plausible low α/β ratio (≤8 Gy). The reoxygenation model provided a performance comparable to the LQL model for low-risk prostate cancer and slightly inferior for intermediate-risk. **Conclusions:** This suggests that the observed reduction in tumor control may not necessarily be a failure of the LQ formalism but, rather, a consequence of oxygen dynamics associated with ultra-hypofractionated schedules. Nonetheless, neither this nor our previous work can provide insight into the driving mechanism and should only be interpreted as showing that both hypotheses are compatible with the clinical data.

## Simple Summary

Several clinical trials have shown poor tumor control of prostate cancer when treated with hypofractionated high-dose-rate brachytherapy (HDR-BT), which is in contradiction with classical studies that showed that prostate cancer is very sensitive to hypofractionation. The origin of this poor response is not yet fully understood. In this work, we have investigated whether hypoxia and reoxygenation can be behind such poor control for extreme hypofractionation by analyzing a large dataset of response to HDR-BT with an advanced reoxygenation model. These results may assist in the design of radical HDR-BT treatments.

## 1. Introduction

The response of prostate cancer to radiotherapy is probably the most studied case in the radiobiological modeling literature [1,2,3]. The clear consensus is that the α/β ratio of prostate cancer is very low (typically in the 1–4 Gy range), and this tumor is very sensitive to fractionation. In recent years, extreme hypofractionation has become widely used to treat prostate cancer by using either stereotactic body radiotherapy (SBRT) or high-dose-rate brachytherapy (HDR-BT). HDR-BT is a highly conformal radiotherapy technique that delivers radiation directly within the prostate using temporary radioactive sources (usually Ir192 or Co60). HDR-BT remains a highly effective and established monotherapy option for well-selected patients with low- and intermediate-risk localized prostate cancer, achieving excellent clinical outcomes while significantly reducing overall treatment time through extreme hypofractionated schedules compared to external radiotherapy. Conversely, for high-risk patients, HDR-BT is generally not indicated as a monotherapy but remains a crucial component as a dose-escalation boost in combination with external radiotherapy and androgen deprivation therapy. While SBRT treatments reach 10 Gy per fraction, HDR-BT treatments can reach >20 Gy in a single fraction. Interestingly, those ultra-hypofractionation studies have reported a significant reduction in tumor control (<70%), which seems to be in contradiction with the classical paradigm of a low α/β ratio for prostate cancer.

Guirado et al. were the first group to analyze the response of prostate cancer to hypofractionated HDR-BT, proposing a large α/β ratio (∼23 Gy) to explain the poor control achieved with single-fraction HDR-BT treatments [4]. This value is in conflict with many radiobiological studies on classical radiotherapy and SBRT that support a low α/β for prostate cancer. Therefore, they also argued that the linear-quadratic (LQ) model may not be adequate for very large doses per fraction. The latter proposal was further explored in a recent publication from our group [5]. We curated a large dataset of the response to HDR-BT, including multiple fractionation schemes, and analyzed it with different models, showing that the LQL model of dose-response [6], which softens the damage predicted by the LQ model at large doses per fraction, was capable of describing the clinical response to single-fraction HDR-BT treatments while keeping a low α/β ratio.

While the LQ model has long been questioned [7] at large doses per fraction, which may be behind the poor response observed for single-fraction HDR-BT, other biophysical factors could also play a role. In particular, reoxygenation (or the lack thereof for hypofractionated schemes) has been investigated in the context of prostate cancer, starting with the seminal work by Nahum et al. [8] (aiming at conventional radiotherapy), and could play a role in the poor response to single-fraction HDR-BT. In Ref. [5], we also performed a preliminary analysis of the effect of reoxygenation by using a simple LQ+reoxygenation model [9] but found no improvement over the simple LQ model when including reoxygenation. In this work, we have extended the analysis of the role of hypoxia and reoxygenation by using a more comprehensive model originally introduced by Jeong et al. [10]. These authors modeled reoxygenation through the shift of cells between three compartments with different oxygenation levels and varying radiosensitivities (modeled through OERs). Oxygen plays a complex role in the response to radiation, affecting not only the radiosensitivity of tumor cells (oxygen enhancement ratios, OERs) but also the induction and chemical stabilization of complex DNA lesions, including clustered DNA damage [11]. While these intricate effects or the complex heterogeneity in the tumor are not fully captured in a macroscopic compartmental model, this framework has nonetheless been shown to be able to describe the clinical dose-response data [12,13].

## 2. Materials and Methods

### 2.1. Clinical Dataset

Building upon our previous work [5], this study uses the same clinical dataset, which allows for a systematic intercomparison with models analyzed in that work (LQ, LQL and Stavrev et al. reoxygenation). The dataset contains dose-response data (local control at five years) for 21 schedules (1633 patients) for low-risk (LR) and 23 schedules (1606 patients) for intermediate-risk (IR) prostate cancer, with doses per fraction ranging from 6 Gy to 21 Gy.

### 2.2. Radiobiological Modeling

#### 2.2.1. MSK Model

In this work, we have adapted the response model of Jeong et al. [10] to analyze the clinical response of prostate cancer to HDR-BT. We will refer to this model as the MSK model, in reference to the institution of the authors. This model is a compartmental model that splits tumor cells into three different compartments according to their oxygenation levels: (*P*) well-oxygenated cells (which are proliferative), (*I*) intermediate-oxygenated cells, and (*H*) hypoxic cells. Radiation damage is modeled using the LQ model and affects cells differently according to their oxygenation status (with well-oxygenated cells being the most sensitive to radiation due to the oxygen enhancement ratio, OER). Damaged cells die when trying to undergo mitosis in the *P* compartment, which creates vacancies in the *P* compartment that trigger a shift from poorly oxygenated to well-oxygenated compartments, thereby reoxygenating the tumor. This model has been validated with clinical data [12,13]. In particular, in Ref. [12], the authors used the model to describe the dose-response of a large dataset of NSCLC.

For further details, we refer the reader to the original publication, but for completeness, we present the main equations of the model here. $N_{i}^{j}$ refers to cells in state *i* belonging to the *j* compartment, with *i* = (*v*, *d*) referring to those that are viable (*v*) and those that are doomed due to radiation damage (*d*), and with *j* = (*P*, *I*, *H*) referring to the proliferative (oxic), intermediate, and hypoxic compartments, respectively. In differential form, the model can be written as:

(1) $$\begin{array}{cc} \frac{dN_{v}^{P}}{dt} & =\left(\frac{f_{\mathrm{pro}}\ln\left(2\right)}{T_{C}}\right)N_{v}^{P} \end{array}$$

(2) $$\begin{array}{cc} \frac{dN_{d}^{P}}{dt} & =\left(\frac{\left(2k_{m}-1\right)f_{\mathrm{pro}}\ln\left(2\right)}{T_{C}}\right)N_{d}^{P} \end{array}$$

(3) $$\begin{array}{cc} \frac{dN_{v}^{I}}{dt} & =0 \end{array}$$

(4) $$\begin{array}{cc} \frac{dN_{d}^{I}}{dt} & =0 \end{array}$$

(5) $$\begin{array}{cc} \frac{dN_{v}^{H}}{dt} & =-\left(\frac{\ln(2)}{T_{h}}\right)N_{v}^{H} \end{array}$$

(6) $$\begin{array}{cc} \frac{dN_{d}^{H}}{dt} & =-\left(\frac{\ln(2)}{T_{h}}\right)N_{d}^{H} \end{array}$$

Notice that the cells in the *I* compartment have no dynamics, as they cannot proliferate and do not die during mitosis (they only do so if/when they move to the proliferative compartment). Viable cells in the *P* compartment proliferate, and doomed cells in *P* die during mitosis (notice that $(2k_{m}-1)<0$). The cells in the *H* compartment die due to severe hypoxia. Each radiation dose acts as an impulse to the differential equations, moving cells from viable to doomed in each compartment for each radiation dose, *d*, delivered at time $t_{D}$, as:

(7) $$\begin{array}{cc} N_{v}^{P}\left(t_{d}\right) & \to N_{v}^{P}\left(t_{d}\right)\exp\left(-\alpha d-\beta d^{2}\right) \end{array}$$

(8) $$\begin{array}{cc} N_{d}^{P}\left(t_{d}\right) & \to N_{d}^{P}\left(t_{d}\right)+\left(1-\exp\left(-\alpha d-\beta d^{2}\right)\right)N_{v}^{P}\left(t_{d}\right) \end{array}$$

(9) $$\begin{array}{cc} N_{v}^{I}\left(t_{d}\right) & \to N_{v}^{I}\left(t_{d}\right)\exp\left(-\frac{\alpha }{{\mathrm{OER}}_{I}}d-\frac{\beta }{{\mathrm{OER}}_{I}^{2}}d^{2}\right) \end{array}$$

(10) $$\begin{array}{cc} N_{d}^{I}\left(t_{d}\right) & \to N_{d}^{I}\left(t_{d}\right)+\left(1-\exp\left(-\frac{\alpha }{{\mathrm{OER}}_{I}}d-\frac{\beta }{{\mathrm{OER}}_{I}^{2}}d^{2}\right)\right)N_{v}^{I}\left(t_{d}\right) \end{array}$$

(11) $$\begin{array}{cc} N_{v}^{H}\left(t_{d}\right) & \to N_{v}^{H}\left(t_{d}\right)\exp\left(-\frac{\alpha }{{\mathrm{OER}}_{H}}d-\frac{\beta }{{\mathrm{OER}}_{H}^{2}}d^{2}\right) \end{array}$$

(12) $$\begin{array}{cc} N_{d}^{H}\left(t_{d}\right) & \to N_{d}^{H}\left(t_{d}\right)+\left(1-\exp\left(-\frac{\alpha }{{\mathrm{OER}}_{H}}d-\frac{\beta }{{\mathrm{OER}}_{H}^{2}}d^{2}\right)\right)N_{v}^{H}\left(t_{d}\right) \end{array}$$

The capacities of compartments *P* and *I* are finite and given by:

(13) $$\begin{array}{cc} Q_{P} & =\frac{\mathrm{GF}}{f_{\mathrm{pro}}}N_{\mathrm{tot}} \end{array}$$

(14) $$\begin{array}{cc} Q_{I} & =\left(1-\mathrm{GF}\left(\frac{1}{f_{\mathrm{pro}}}+\left(1-\frac{T_{C}}{T_{D}\mathrm{GF}}\right)\frac{T_{h}}{T_{C}}\right)\right)N_{\mathrm{tot}} \end{array}$$

At the beginning of the simulation, the tumor contains $N_{\mathrm{tot}}$ cells, all of which are viable. Cells are assigned to compartment *P* until it is filled, then to compartment *I* until filled, and then to compartment *H* (if necessary). The death of doomed cells in the *P* compartment creates vacancies that are instantly filled by cells (if any) shifting from the *I* compartment (both viable and doomed, according to their ratio in the *I* compartment) and the *H* compartment (if necessary). The shift from *I* to *P* creates vacancies in the *I* compartment that are instantly filled by cells (if any) moving from the H compartment. This compartmental reorganization reoxygenates the tumor.

The model also includes dead cells, which are eventually removed through lysis. However, dead cells do not contribute to the occupancy of the compartments (because they no longer consume oxygen); therefore, they do not affect the dynamics of viable and doomed cells or the calculation of tumor control probabilities (the endpoint modeled in this work) and were excluded from this analysis (they would, however, affect the dynamics of tumor volumes and should be included if one is modeling that aspect).

A list of the parameters of the model is presented in Table 1.

#### 2.2.2. Tumor Control Probability and EQD2

The tumor control probability (TCP) was modeled using a logistic function of the equivalent dose in 2 Gy fractions, EQD2, with parameters $D_{50}$ and $\gamma_{50}$ controlling the dose yielding 50% control and the slope of the dose-response curve, respectively:

(15) $$\mathrm{TCP}=\frac{1}{1+{\left(\frac{D_{50}}{\mathrm{EQD}2}\right)}^{4\gamma_{50}}}$$

The EQD2, was numerically obtained from the model, following [12]. To this end, a reference treatment delivering 150 fractions daily of 2 Gy is simulated, and the number of viable cells at the end of each fraction is saved. These values are then interpolated using a spline function. Finally, in order to calculate the EQD2 of a given schedule, the specific treatment protocol is simulated, and the number of viable cells at the end of the treatment is saved and mapped to its corresponding EQD2 value using the generated spline.

### 2.3. Statistical Methods, Parameter Values, and Implementation

The model and methods were implemented in Matlab 2024b (The Mathworks, Natick, MA, USA). The fitting methodology and statistical tools employed for this dataset are presented in detail in [5,14]. The model was fitted to the clinical data using the maximum likelihood estimation (minimizing the function −ln*L*, where *L* is the likelihood), and confidence intervals for the best-fitting parameter values were computed using the profile likelihood methodology. The Akaike Information Criterion with sample size correction (${\mathrm{AIC}}_{c}$) was used to evaluate model performance in comparison to other investigated models. Additionally, the $\chi^{2}$ test was used to evaluate the goodness of fit. To investigate the predictive performance of the model, a k-fold (*k* = 2) cross-validation was employed. For this purpose, the datasets for LR and IR were split into two subsets; one of them was used for training and the other for validation. To ensure the representation of extreme hypofractionations, we included the constraint that each subset must contain at least two of the single-fraction treatments. This process was repeated 50 times to generate robust statistics.

Several parameters were fixed to values established in the literature. In the cell dynamics model, a value of $N_{\mathrm{tot}}=10^{10}$ was assumed following [10], as this parameter was found to be non-significant for the primary objectives of the analysis. The tumor growth fractions (GF) and doubling times ($T_{D}$) were taken from Zharinov et al. [15], who reported median growth fractions (Ki-67) of 0.083 and 0.116 and doubling times of 34.83 and 30.43 months for tumors that can be classified as LR and IR, respectively.

Ljungkvist et al. [16] investigated the cell loss half-time ($T_{h}$) for several types of cancer, reporting values in the range 17–49 h. Given the high degree of heterogeneity expected within the hypoxic compartment, a death rate corresponding to the lower limit of reported values was deemed appropriate; thus, $T_{h}$ was set to 48 h as in [12]. Finally, the oxygen enhancement ratio for the hypoxic compartment (${\mathrm{OER}}_{H}$) was set to 1.37 according to Chan et al. [17], and $T_{C}$ was set to 48 h according to [18].

The value of α was fixed at 0.15 Gy^−1^ [14]. While α plays an important role in a mechanistic model like the MSK, particularly if fitting TCPs based on the viable number of cells and the LQ-Poisson formulation, the fits of a phenomenological logistic model like Equation (15) are relatively insensitive to α. This is because a change in $D_{50}$ can compensate for variations in α (this was confirmed by the sensitivity analysis).

Furthermore, the parameter space was constrained to ensure biologically and physically plausible solutions (with special emphasis on avoiding negative cell populations arising from Equations (13) and (14) and to improve computational convergence. The constraint ${\mathrm{OER}}_{I}\geq {\mathrm{OER}}_{H}$ was enforced following [12]. The α/β ratio was constrained to relatively low values (≤8 Gy as in [5], corresponding to 95% confidence intervals reported in [14]) to specifically check whether reoxygenation can explain the response to extreme hypofractionation while being in agreement with the reported low α/β for prostate cancer.

Overall, the number of free parameters for model fitting was reduced to six: α/β, $k_{m}$, ${\mathrm{OER}}_{I}$ and $f_{\mathrm{pro}}$ (MSK model), along with $D_{50}$ and $\gamma_{50}$ (logistic TCP model). The constraints used during model fitting are shown in Table 2.

We used the Sobol methodology to investigate the parametric sensitivity of the model, computing first- and total-order Sobol sensitivity indices by using the methodology of Saltelli et al. [19]. Sensitivity was investigated in a hypercube covering ±10% of the best-fitting values (or fixed values), sampling $N=10^{5}$ combinations of parameters. We used reparametrization to handle the constraints above imposed by Equations (13) and (14), defining $p_{1}=\mathrm{GF}/f_{\mathrm{pro}}$ and $p_{2}=T_{h}/T_{h}^{\mathrm{MAX}}$, where $T_{h}^{\mathrm{MAX}}$ is the maximum value of $T_{h}$ such that $Q_{I}\geq 0$ (therefore, a function of GF, $f_{\mathrm{pro}}$, $T_{C}$ and $T_{D}$).

## 3. Results

**Figure 1:**
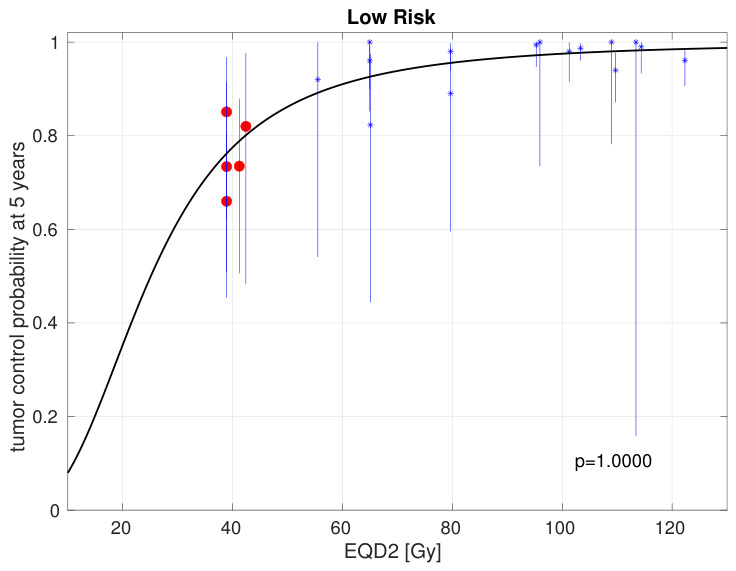

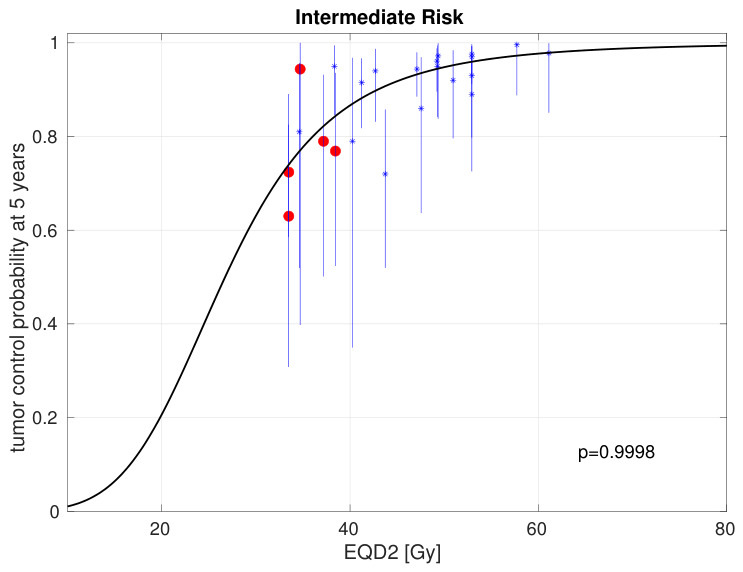
The best fits of the MSK reoxygenation model to dose–response data for prostate cancer treated with HDR-BT, separated into low-risk ((**top**) panel) and intermediate-risk ((**bottom**) panel). Clinical data (*) and 95% confidence intervals (bars), and modeled curves (solid lines). Single fraction schedules are highlighted as red circles. The *p* value of the $\chi^{2}$ test is reported for each fit.

In Figure 1, we present the best fits of the model to the dose-response dataset, independently for LR and IR patients. Single fraction schedules are highlighted in Figure 1. In Table 3, we present the best-fitting parameters for LR and IR, and the parameters describing the goodness-of-fit (−ln*L*, ${\mathrm{AIC}}_{c}$), and the *p* value obtained from the $\chi^{2}$ test. Also, 95% CIs are presented in the table and in more detail in Supplementary Figure S1. Because the computation of CI with the profile likelihood method is computationally demanding, we have restricted this computation, prioritizing the parameters of the MSK model (which characterize the mechanistic drivers of the response) over the phenomenological parameters of the logistic TCP.

The results of the Sobol sensitivity analysis, first-order and total-order indices for each parameter, are presented in Table 4. The results of the *k* = 2 cross-validation are presented in Table 5. In this case, we report the mean and standard deviation of the *p* values obtained from the $\chi^{2}$ test for the training and validation datasets (50 experiments).

## 4. Discussion

Several HDR-BT clinical trials have shown a significant loss of tumor control when treating prostate cancer with extremely hypofractionated protocols (∼20 Gy in a single fraction) [20,21,22,23,24,25,26], which is inconsistent with the widely assumed low α/β ratio of prostate cancer and the LQ model. Among the potential mechanisms underlying this behavior is the failure of the LQ model at large doses per fraction [7,27], with some studies suggesting that it should be replaced by models like the LQL in this regime. Hypoxia and reoxygenation have also been proposed to play a role in the response of prostate cancer [8].

In a recent publication [5], we analyzed the response of prostate cancer to HDR-BT and found that the LQL model could successfully fit the observed dose-response data at large doses per fraction while preserving a low intrinsic α/β value. This seemed to point toward a failure of the standard LQ model in the high-dose per fraction regime. In that work, we also performed a preliminary analysis of reoxygenation using a simple formulation but found that it could not describe the clinical data as well as the LQL model. In this follow-up work, we have performed a deeper investigation of the role of hypoxia and oxygenation in the response to HDR-BT ultra-hypofractionation by using a more comprehensive model, which we call here the MSK model, originally introduced by Jeong et al. [10] and well validated for clinical dose-response analysis [12]. This study was performed on the multi-institutional dataset curated in ref. [5], which contains 21 schedules (1633 patients) for LR and 23 schedules (1606 patients) for IR. A limitation of this approach is that it may suffer from inter-study heterogeneities (different studies may use different margins, different dose constraints on the PTV, different dose calculation algorithms, etc.).

Our current findings show that the LQ model and a low α/β value are consistent with the observed loss of tumor control in ultra-hypofractionated protocols when reoxygenation is accounted for with the MSK model. Fits to LR and IR data yielded *p*-values ($\chi^{2}$ test) of 1.0000 and 0.9998, respectively. While these high *p*-values highlight the goodness of the fits, as seen in Figure 1, they may also indicate an overestimation of experimental uncertainties. The origin of a potential overestimation may be two-fold: (i) first, our approach to correcting the dataset for differences in follow-up (detailed in [5]) may have been too conservative and led to an overestimation of some uncertainties; (ii) second, we used binomial statistics to model the uncertainties of control data, whereas the original tumor control rates are described by Kaplan-Meier statistics, and the binomial formulation can overestimate the uncertainties for schedules with small patient cohorts. Interestingly, previous clinical studies employing the MSK model also showed very high *p*-values [12]. The best-fitting parameters reported in Table 3 suggest a more prominent role for hypoxia for IR than for LR (34% of cells in the oxic proliferative compartment at baseline for IR versus 92% for LR), which is supported by experimental evidence [28], yet a faster reoxygenation for LR, shown by the value of $k_{m}$. Despite the higher relative presence of cells in the intermediate/hypoxic compartments for IR, the optimization was unable to find an optimal solution characterized by accelerated reoxygenation kinetics (lower $k_{m}$ value). Why this is the case is not fully understood by the authors but may be related to the model parameters that were constrained.

The performance of the cross-validation merits closer inspection. Overall, the average *p*-values for the training datasets were 0.917±0.064 and 0.873±0.114 for LR and IR, respectively, while the corresponding values for the validation datasets were 0.516±0.298 and 0.385±0.358 (mean ± standard deviation across 50 random splits). A detailed analysis of the cross-validation results showed that a single specific trial strongly conditions these validation outcomes: the schedule reported by Strouthos et al. [22]. This schedule has an unusually long overall treatment time (OTT) of 42 days and a large number of patients (meaning that the experimental TCP has narrow uncertainties). For the LR cohort, 10 out of the 50 cross-validations yielded p< 0.05 in the validation dataset, and in every single one of these instances, the Strouthos schedule was part of the validation dataset. For the IR cohort, 18 out of 50 experiments failed the *p*-test, with the Strouthos schedule being part of the validation dataset in 16 of them. Due to its long OTT, the model’s predicted outcome becomes highly sensitive to tumor proliferation. Since no other schedule in our dataset has such long OTT values, the model cannot robustly optimize the proliferation parameters unless the Strouthos schedule is included in the training dataset. The problem is exacerbated by the large number of patients (narrow TCP uncertainties). For reference, when this specific schedule was part of the training dataset, *p*-values on the validation dataset were 0.661 ± 0.150 and 0.614 ± 0.309 for LR and IR, respectively.

These results were compared to those reported in [5] for the LQL model (notice that we used exactly the same dataset in both studies to facilitate the comparison). The ${\mathrm{AIC}}_{c}$ values obtained in this work for the MSK model were 91.34 (LR) and 108.06 (IR), versus 89.25 (LR) and 103.41 (IR) obtained for the LQL; the *p*-values obtained in this work for the MSK model were 1.0000 (LR) and 0.9998 (IR), versus 1.0000 (LR) and 1.0000 (IR) obtained for the LQL. Consequently, the hypoxia/reoxygenation model is statistically comparable to the LQL model for LR ($\Delta {\mathrm{AIC}}_{c}≃$ 2.1) and moderately inferior for IR ($\Delta {\mathrm{AIC}}_{c}≃$ 4.5). In information theoretic model selection, differences in ${\mathrm{AIC}}_{c}$ above 10 are typically required to state the superiority of a given model [29]. Notice that the ${\mathrm{AIC}}_{c}$ penalizes the number of free parameters, and the MSK model inherently contains more parameters than the LQL model. However, because we have fixed several parameters according to the literature, the number of free parameters of both models is the same.

Elucidating the underlying mechanisms responsible for the significant loss of local control when delivering extremely hypofractionated HDR-BT seems to be of paramount importance of designing effective clinical protocols. Our present work shows that the MSK reoxygenation model provides a data-compatible explanation without departing from the standard LQ formalism, especially for LR data. However, when our current findings are evaluated alongside our previous study, the clinical data alone do not allow us to definitively separate the reoxygenation hypothesis from the hypothesis of LQL behavior at large doses per fraction. Nonetheless, neither this nor our previous work can provide insight into the driving mechanism, and it should only be interpreted as showing that both hypotheses are compatible with the clinical data (even though the AIC analysis slightly favors the LQL model). More clinical data are needed to shed light on the mechanism underlying the response of prostate cancer to ultra-hypofractionation. In particular, if hypoxia and reoxygenation are the primary drivers of this behavior rather than an intrinsic LQL response, an analysis of the role of pre-treatment tumor hypoxia (measured with functional imaging, for example) might show a far larger drop-off in tumor control between hypoxic and oxic tumors when treated with extreme hypofractionation than when treated with less radical fractionations. While the impact of hypoxia on conventional fractionation is well documented, with Milosevic et al. [30] reporting a difference in local control of ∼7% between oxic and hypoxic tumors, a similar study has not been performed for extreme hypofractionation as far as we know.

## 5. Conclusions

In this work, we have shown that the observed loss of tumor control in ultra-hypofractionated HDR-BT protocols for prostate cancer is compatible with the LQ model and an intrinsic low α/β value, provided that hypoxia and reoxygenation kinetics are taken into account. Nevertheless, an alternative LQL-like behavior at large doses per fraction cannot be excluded as the driver of this drop-off in control. More clinical data are needed to elucidate the mechanism/mechanisms driving the response of prostate cancer to ultra-hypofractionation. Overall, the implementation of the MSK model presented here offers a valuable framework for interpreting the response of prostate cancer to ultra-hypofractionated HDR-BT protocols. This model and analysis may serve as useful benchmarks to assist in the design of future protocols.

## Figures and Tables

**Table 1:** Radiobiological parameters of the MSK compartmental model. For parameters that have been set to a fixed value, we provide such values (see Section 2.3 for more details), while parameters that have been fitted to the experimental data are labeled as “Free parameters”.

Parameter	Description	Value (Unit)
$f_{\mathrm{pro}}$	Proliferating fraction in the *P* compartment	Free parameter
$T_{C}$	Cell cycle time	48 h
$T_{D}$	Tumor doubling time	34.83 months (LR), 30.43 months (IR)
GF	Growth fraction of the tumor	0.083 (LR), 0.116 (IR)
$k_{m}$	Mitotic survival probability of doomed cells	Free parameter
$T_{h}$	Half-life of cells in the hypoxic compartment *H*	48 h
α	Linear radiosensitivity parameter for oxic cells	0.15 Gy^−1^
α/β	α/β ratio for oxic cells	Free parameter
${\mathrm{OER}}_{I}$	Oxygen Enhancement Ratio for compartment *I*	Free parameter
${\mathrm{OER}}_{H}$	Oxygen Enhancement Ratio for compartment *H*	1.37
$N_{\mathrm{tot}}$	Total initial number of cells	10^10^

**Table 2:** Optimization constraints and search space for the model parameters.

Parameter	Minimum	Maximum
$D_{50}$ (Gy)	10	150
$\gamma_{50}$	0.1	4
$k_{m}$	0	0.4
α/β (Gy)	0.1	8
${\mathrm{OER}}_{I}$	1.37	2.5
$f_{\mathrm{pro}}$	0	1

**Table 3:** Best fits obtained with the MSK reoxygenation model to dose–response data for prostate cancer treated with HDR-BT, separated by risk (low, LR, and intermediate risk, IR). The table shows the best-fitting parameters, maximum likelihood and ${\mathrm{AIC}}_{c}$, and *p* value of the $\chi^{2}$ test. The symbols * and + are used to indicate that the parameter reached the edge of the constraint window, or that the parameter reaches the limit of positivity of cells given by Equation (14).

	Low Risk (LR)		Intermediate Risk (IR)	
	Values	95% CI	Values	95% CI
α/β [Gy]	0.96	[0.84, 8 *]	8.0 *	[5.06, 8 *]
${\mathrm{OER}}_{I}$	2.5 *	[1.93, 2.5 *]	2.5 *	[1.61, 2.5 *]
$k_{m}$	0.0 *	[0.0 *, 0.40 *]	0.40 *	[0.24, 0.4 *]
$f_{\mathrm{pro}}$	0.09	[0.090 ^+^, 0.093]	0.34	[0.28, 0.48]
$D_{50}$ [Gy]	25.11	-	26.73	-
$\gamma_{50}$	0.66	-	1.16	-
−lnL	36.67	-	45.40	-
${\mathrm{AIC}}_{c}$	91.34	-	108.06	-
*p* ($\chi^{2}$ test)	1.0000	-	0.9998	-

**Table 4:** Parametric sensitivity analysis of the model: first-order (*S*) and total-order ($S_{T}$) Sobol sensitivity indices.

	LR		IR	
Parameters	S	$S_{T}$	S	$S_{T}$
$D_{50}$	0.042	0.088	0.293	0.909
$\gamma_{50}$	0.006	0.020	0.000	0.387
$p_{1}$	0.399	0.563	0.001	0.002
$T_{D}$	0.000	0.000	0.000	0.000
$T_{C}$	0.002	0.004	0.000	0.004
$p_{2}$	0.266	0.422	0.000	0.000
$k_{m}$	0.001	0.003	0.012	0.012
α	0.009	0.020	0.000	0.009
α/β	0.003	0.006	0.000	0.129
${\mathrm{OER}}_{I}$	0.053	0.101	0.047	0.2213
${\mathrm{OER}}_{H}$	0.000	0.000	0.000	0.000
$f_{\mathrm{pro}}$	0.002	0.004	0.000	0.003

**Table 5:** Results of the *k* = 2 cross-validation: mean and standard deviation of the *p* values obtained from the $\chi^{2}$ test for the training and validation datasets (50 experiments), for LR and IR.

LR		IR	
Training	Validation	Training	Validation
0.917 ± 0.064	0.516 ± 0.298	0.873 ± 0.114	0.385 ± 0.358

## Data Availability

Code and supporting data are available from the Zenodo repository: https://doi.org/10.5281/zenodo.19923987.

## Supplementary Materials

The following supporting information can be downloaded at: https://www.mdpi.com/article/10.3390/cancers18122007/s1, Supplementary Figure S1: Illustration of the calculation of 95% confidence intervals of several parameters through the profile likelihood method. The gray areas show the constraints used for each parameter.

## Abbreviations

The following abbreviations are used in this manuscript:

HDR-BT	high-dose-rate brachytherapy
LQ	linear-quadratic
LQL	linear-quadratic-linear
LR	low risk
IR	intermediate risk
SBRT	stereotactic body radiotherapy
AIC	Akaike information criterion
MSK	Memorial Sloan Kettering
