# Supplementary material for: Analysis of the Response of Prostate Cancer to Ultra-Hypofractionated High-Dose-Rate Brachytherapy: The Role of Hypoxia and Reoxygenation
Source: Cancers (Basel). 2026 Jun 21;18(12):2007. doi: 10.3390/cancers18122007 (PMC13297399; doi:10.3390/cancers18122007)
Supplement: Supplementary file 1 [file cancers-18-02007-s001.zip › cancers-4333747-supplementary.pdf]

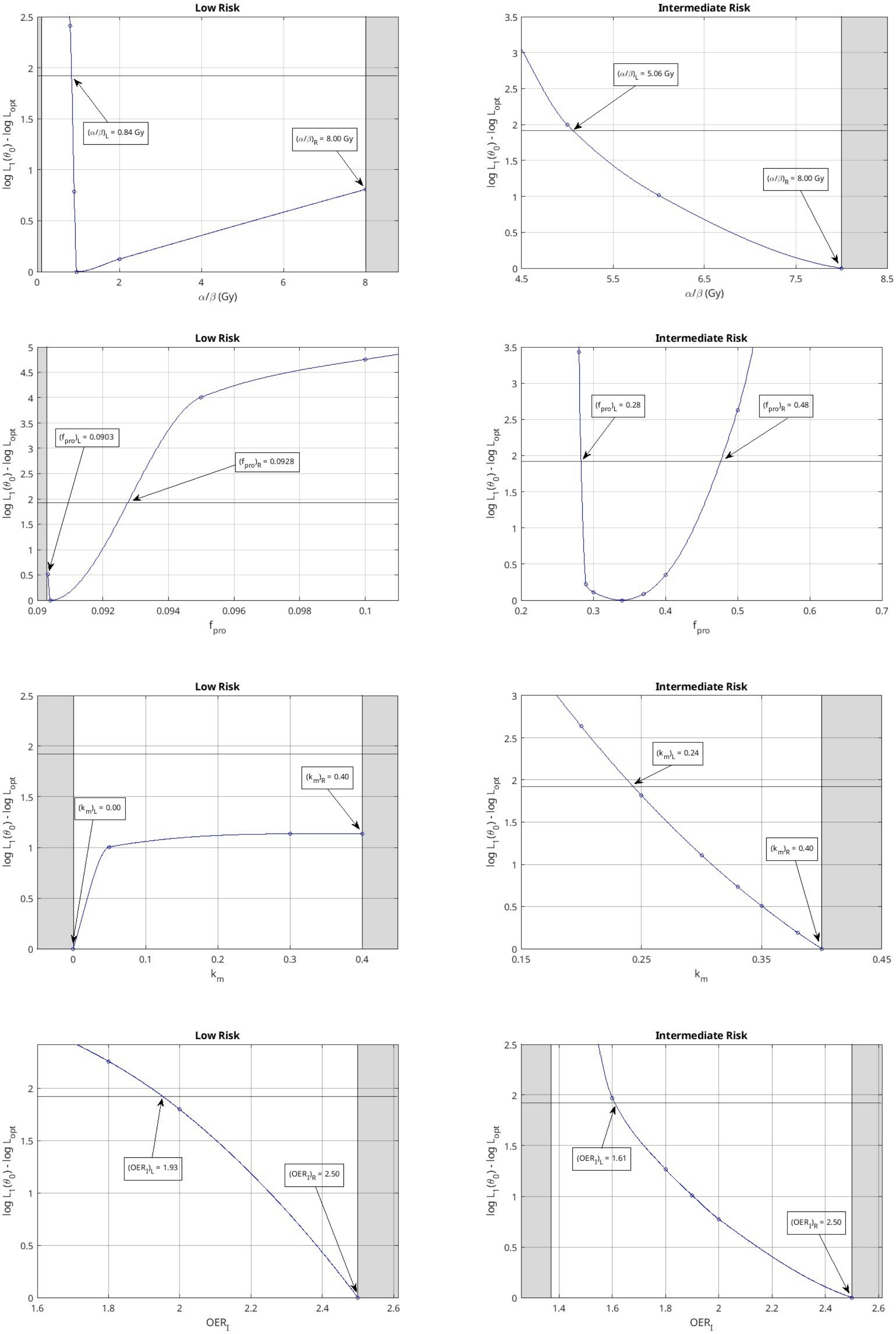

Supplementary Figure S1: Illustration of the calculation of 95% confidence intervals of several parameters through the profile likelihood method. The gray areas shown the constraints used for each parameter.
